# Performance of computer-aided detection of pulmonary nodules in low-dose CT: comparison with double reading by nodule volume

**DOI:** 10.1007/s00330-012-2437-y

**Published:** 2012-07-20

**Authors:** Yingru Zhao, Geertruida H. de Bock, Rozemarijn Vliegenthart, Rob J. van Klaveren, Ying Wang, Luca Bogoni, Pim A. de Jong, Willem P. Mali, Peter M. A. van Ooijen, Matthijs Oudkerk

**Affiliations:** 1 Center for Medical Imaging – North East Netherlands, Department of Radiology, University of Groningen/University Medical Center Groningen, P.O. Box 30.001, 9700RB Groningen, the Netherlands; 2 Department of Epidemiology, University of Groningen/University Medical Center Groningen, P.O. Box 30.001, 9700RB Groningen, the Netherlands; 3 Department of Pulmonology, Lievensberg Hospital, P.O. Box 135, 4600AC Bergen op Zoom, the Netherlands; 4 CAD Group, Siemens Medical Solutions USA, Inc., 51 Valley Stream Parkway, Malvern, PA 19355 USA; 5 Department of Radiology, University Medical Center Utrecht, University of Utrecht, P.O. Box 85500, 3508GA Utrecht, the Netherlands

**Keywords:** Computer-aided detection, Multi-detector computed tomography, Pulmonary nodules, Low dose, Volumetry

## Abstract

**Objective:**

To evaluate performance of computer-aided detection (CAD) beyond double reading for pulmonary nodules on low-dose computed tomography (CT) by nodule volume.

**Methods:**

A total of 400 low-dose chest CT examinations were randomly selected from the NELSON lung cancer screening trial. CTs were evaluated by two independent readers and processed by CAD. A total of 1,667 findings marked by readers and/or CAD were evaluated by a consensus panel of expert chest radiologists. Performance was evaluated by calculating sensitivity of pulmonary nodule detection and number of false positives, by nodule characteristics and volume.

**Results:**

According to the screening protocol, 90.9 % of the findings could be excluded from further evaluation, 49.2 % being small nodules (less than 50 mm^3^). Excluding small nodules reduced false-positive detections by CAD from 3.7 to 1.9 per examination. Of 151 findings that needed further evaluation, 33 (21.9 %) were detected by CAD only, one of them being diagnosed as lung cancer the following year. The sensitivity of nodule detection was 78.1 % for double reading and 96.7 % for CAD. A total of 69.7 % of nodules undetected by readers were attached nodules of which 78.3 % were vessel-attached.

**Conclusions:**

CAD is valuable in lung cancer screening to improve sensitivity of pulmonary nodule detection beyond double reading, at a low false-positive rate when excluding small nodules.

**Key Points:**

*• Computer-aided detection (CAD) has known advantages for computed tomography (CT).*

*• Combined CAD/nodule size cut-off parameters assist CT lung cancer screening.*

*• This combination improves the sensitivity of pulmonary nodule detection by CT.*

*• It increases the positive predictive value for cancer detection.*

## Introduction

The rapid development of multi-detector CT (MDCT) has increased the amount of data for radiologists to analyse. Reviewer’s fatigue increases the risk of false-negative diagnosis due to perceptual error. In addition, although the introduction of low-dose CT in lung cancer screening protocols was found effective in detecting peripheral lung cancers at an early stage [1–4], it may be more difficult for screening radiologists to find lesions in case of increased image noise due to low dose and thin slice thickness. To reduce the number of missed lesions, double reading has been recommended [5]. Previous studies have found that more pulmonary nodules are detected by double reading than by single reading [5, 6]. However, double reading is not widely used in clinical routine because of limited human resources and cost-effectiveness [7, 8].

Computer-aided detection (CAD) of pulmonary nodules may help address this problem by being utilised as an assistant reader [9–13]. A significant improvement in sensitivity was shown in pulmonary nodule detection, albeit at the disadvantage of a large increase in false-positive (FP) findings. Previous studies have found that CAD increases the sensitivity of pulmonary nodule detection compared to that of single human reading [14, 15]. In one rather small study, true-positive (TP) findings identified with the aid of CAD complemented radiologists’ TP findings to a greater extent than those contributed by second readers [16].

In lung cancer screening, small pulmonary nodules are extremely common findings. Previous studies using low-dose CT for early detection of asymptomatic lung cancer in populations at risk reported that more than 95 % of nodules 10 mm or smaller were benign [1, 2, 17]. Available data indicate that less than 1 % of very small (less than 5 mm, corresponding to 65.4 mm^3^) nodules were malignant [2, 18, 19]. Therefore, a size cut-off in CAD could be more efficient in helping radiologists make diagnoses. In recent years, volume instead of diameter has become an important factor to evaluate nodule size and growth. As this measure is more accurate for evaluating growth [20–22], a volume cut-off is likely more precise in distinguishing probably malignant and probably benign nodules.

The purpose of our study was to assess the performance of CAD for detection of pulmonary nodules as a complementary tool in a large-scale, low-dose CT lung cancer screening study compared to double reading, with stratification according to nodule volume. Double reading is the original design of nodule evaluation in our lung cancer screening trial. The hypothesis of the current study was that CAD increases sensitivity of lung nodule detection beyond double reading.

## Materials and methods

### Study population

The subjects in this study were participants of the four screening sites of the Dutch–Belgian randomised trial for lung cancer screening (NELSON) by low-dose MDCT. The protocol required participants to be current or former smokers with a smoking history of more than 15 cigarettes/day for longer than 25 years or more than 10 cigarettes/day for longer than 30 years. The NELSON study was approved by the medical ethics committees of all institutions and all participants provided written informed consent [23] that also covered the current analysis. As a side-study of the NELSON project, we randomly selected 400 out of 4,280 baseline CTs from 2005 using a statistical program (SPSS 16.0 for Windows, SPSS, Inc., Chicago, IL, USA).

### CT protocol

At all screening sites 16-detector CT was used (3 Sensation-16, Siemens Medical Solutions, Forchheim, Germany and 1 Mx8000 IDT or Brilliance 16P, Philips Medical Systems, Cleveland, OH, USA). CT of the entire chest was performed, in caudo-cranial direction. CT data were acquired with 16 × 0.75 mm collimation and pitch of 1.3. No intravenous contrast medium was used. Low-dose settings were applied depending on body weight (less than 50 kg, 50–80 kg and greater than 80 kg), with corresponding kVp settings of 80–90, 120 and 140 kVp, to achieve a CT dose index volume of approximately 0.8, 1.6 and 3.2 mGy, respectively. The mAs settings were adjusted accordingly depending on the machine used. To minimise breathing artefacts, CT data acquisition was performed at suspended maximal inspiration after appropriate instructions were given to the subjects. Data were reconstructed at 1.0-mm slice thickness, with 0.7-mm reconstruction increments and soft kernel (Siemens B30 filter, Siemens Medical Solutions, Forchheim, Germany). The Siemens B30 kernel is the standard soft tissue reconstruction kernel. Transversal, 6-mm-thick maximum intensity projections (MIP) reconstructions were used to identify pulmonary nodules.

### Evaluation of CT examinations by double reading

At the time of acquisition (2005), all CT images of the lungs from each examination were independently read by first and second readers (double reading) as part of the NELSON protocol [6, 23]. The first reading was performed by 13 readers (experience in reading thoracic CT varying from 0 to 20 years); the second reading was performed by two readers, each with 6 years of experience. Upon identifying a finding as a pulmonary nodule, volume measurement was performed by the individual readers as part of the double reading. The LungCARE© software package (Leonardo© workstation, Somaris/5 VB10A, Siemens Medical Solutions, Erlangen, Germany) designed to aid readers in measuring and characterising pulmonary nodules was used in addition to visual readings by all readers. Nodule diameter and volume were automatically calculated using this three-dimensional (3D) volumetric assessment tool. In case of inappropriate segmentation, the radiologists could perform manual two-dimensional (2D) measurements using a calliper.

### Lung CAD algorithm

The lung CAD algorithm evaluated in this study was a commercial software version available since 2006 (LungCAD VB10A, Siemens AG Healthcare) [24]. This is an extensively validated CAD software, designed as a multi-step approach aiming to detect parenchymal lesions at high sensitivity and specificity, focusing on solid lesions larger than 3 mm. All CT images were processed by this LungCAD software package to mark potential lesions. The findings were reviewed both as 2D-axial images and 3D rendered views obtained with LungCARE. The MIP reconstruction settings used in LungCAD were equal to those in LungCARE.

### Evaluation of findings by consensus panel

Retrospectively, a consensus panel of two expert radiologists with at least 8 years of experience in reading thoracic CT reviewed the CAD-marked images and the results obtained from double reading were entered into the NELSON management system, and compared the findings in LungCARE [23]. The consensus panel did not search for potential additional nodules. This reference standard was similar to previously reported practices [14, 25]. The consensus panel labelled the findings as “nodule” according to the definitions in the NELSON protocol [23]. Upon identifying a finding as a pulmonary lesion, volume measurement was performed by the consensus panel. Conforming with the image reading protocol used by the readers in the double reading, nodules smaller than 15 mm^3^ were not assessed whereas larger non-calcified solid nodules were classified into three categories based on size (negative nodule, smaller than 50 mm^3^; indeterminate nodule, 50–500 mm^3^; positive nodule, larger than 500 mm^3^) [26]. A cut-off of 50 mm^3^ (4.6 mm diameter) was chosen as previous studies have shown that the possibility of malignancy in these small nodules is negligible [18, 19]. Because consistent volume measurement was not possible in non-/part-solid nodules, the calliper was used to measure the largest axial diameter of these lesions. Non-solid and part-solid nodules with non-solid component at least 8 mm as well as part-solid nodules with solid part larger than 50 mm^3^ were considered indeterminate nodules.

Findings were divided into two groups based on NELSON’s nodule categories: findings that could be excluded from further evaluation and those that needed further evaluation. Findings excluded from further evaluation were subdivided into three sub-groups: negative nodule (smaller than 50 mm^3^), benign lesion or non-lesion. Calcifications and abnormal findings not presenting as nodule shapes, e.g. pleural plaque, fissure thickening or fibrosis, were recorded as benign lesions. A finding was assigned as “non-lesion” if the finding was due to normal anatomy or artefact. Findings needing further evaluation consisted of indeterminate and positive nodules. These findings were subsequently characterised by the consensus panel in terms of location (peripheral or non-peripheral), consistency (solid or non-/part-solid), attachment (intraparenchymal, fissure-attached, vessel-attached or pleural-based), shape (spherical or non-spherical) and edge (smooth or non-smooth) [23, 27]. Nodules were classified as peripheral if the distance to the thoracic wall was less than one third of the total distance from the thoracic wall to the lung hilum, and as non-peripheral otherwise.

### Data analysis

Findings from double reading and from CAD were labelled either as TP, if they were determined by the consensus panel as findings needing further evaluation, or otherwise as FP. Sensitivity for pulmonary nodules from double reading and of CAD findings was calculated using the consensus panel as the reference standard. The FP rate presented with respect to nodule volume (less than or at least 50 mm^3^) was computed as the number of FP detections per CT. Additionally, the positive predictive value of findings detected by CAD was calculated. The probability of detecting pulmonary nodules according to nodule characteristics was tested between CAD and double reading by the McNemar test. All statistical analyses were performed using SPSS 16.0.

## Results

**Fig. 1:**
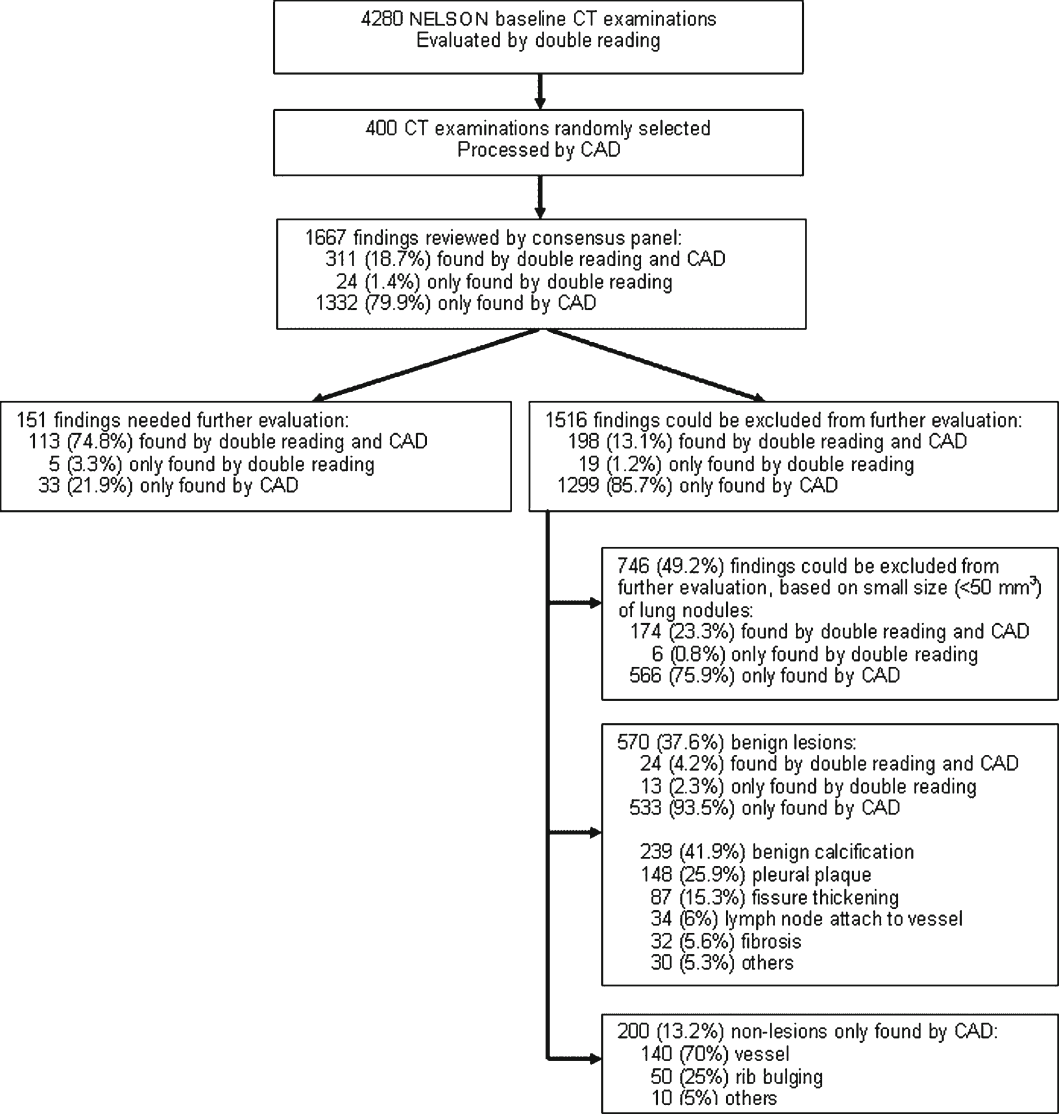
Flow chart of nodule detection and evaluation

The mean age of the 400 participants was 59 ± 6 years (range 51–76 years). On 332 of the 400 baseline CT examinations at least one finding was reported (Fig. 1). A total of 1,667 findings were detected by the readers and CAD system. A total of 90.9 % (*n* = 1,516) of the identified findings could be excluded from further evaluation (Fig. 1). In this study, these findings were considered as FP findings. The FP rate was 3.7 per CT for CAD and 0.5 per CT for readers (Table 1). Excluding small nodules (less than 50 mm^3^) and regarding benign lesions and non-lesions as FP findings only, the FP rate for CAD decreased to 1.9. By using 50 mm^3^ as the cut-off below which pulmonary nodules were disregarded, the positive predictive value of CAD increased from 8.9 to 16.2 %. The positive predictive value of double reading was 35.2 % and 76.1 % for all nodules and nodules larger than 50 mm^3^, respectively.

**Table 1:** Sensitivity, false positive (FP) rate and positive predictive value (PPV) of nodule detection for double reading and CAD in all nodules and nodules larger than 50 mm^3^

	All nodules		Nodules >50 mm^3^	
	Double reading	CAD	Double reading	CAD
Sensitivity (%)	78.1	96.7	78.1	96.7
FP/examination (*n*)	0.5	3.7	0.1	1.9
PPV (%)	35.2	8.9	76.1	16.2

According to the consensus panel, 151 (9.1 %) of 1,667 findings needed further evaluation. Of these 151 nodules, 113 were found both by readers and CAD, and 33 and 5 were found only by CAD or readers, respectively (Fig. 1). The overall sensitivity for potentially significant pulmonary nodules (indeterminate and positive pulmonary nodules) was 78.1 % for readers and 96.7 % for CAD (Table 1).

Table 2 presents an overview of the nodules found by readers or by CAD. Among the 151 indeterminate and positive pulmonary nodules, 76.6 % were located peripherally, 96.7 % were solid nodules, 49.7 % were intraparenchymal and 76.2 % were spherical and 87.4 % were smooth. The median volume of 146 solid nodules was 85.4 mm^3^ (range 50.0–1,672.4 mm^3^). Consistent volume measurement was not possible in the 5 non-/part-solid nodules. CAD was better in detecting most types of nodules, namely peripheral and non-peripheral nodules, solid nodules, intraparenchymal nodules, and spherical and non-spherical nodules. Some differences could not be tested for significance as some cells were empty (for non-/part-solid, vessel-attached, non-smooth and positive nodules).

**Table 2:** Characteristics of 151 pulmonary nodules needing further evaluation, found by CAD and/or double reading

Variable	*n*	Nodules found by		*P* value
		CAD (%)	Double reading (%)	
Location				
Peripheral	116	112 (96.6)	94 (81.0)	<0.01
Non-peripheral	35	34 (97.1)	24 (68.6)	<0.01
Consistency				
Solid	146	143 (97.9)	113 (77.4)	<0.001
Non-/part-solid	5	3 (60.0)	5 (100)	NA
Attachment				
Intraparenchymal	75	73 (97.3)	65 (86.7)	<0.05
Fissure-attached	18	17 (94.4)	17 (94.4)	NS
Vessel-attached	29	29 (100)	11 (37.9)	NA
Pleural-based	29	27 (93.1)	25 (86.2)	NS
Shape				
Spherical	115	111 (96.5)	92 (80.0)	<0.001
Non-spherical	36	35 (97.2)	26 (72.2)	<0.05
Edge				
Smooth	132	130 (98.5)	99 (75.0)	<0.001
Non-smooth	19	16 (84.2)	19 (100)	NA
Volume^a^ (mm^3^)				
50–500	141	138 (97.9)	108 (76.6)	<0.001
>500	5	5 (100)	5 (100)	NA

*NA* not applicable, *NS* not significantly different

^a^For 5 non-/part-solid nodules, volume was not available

**Fig. 2:**
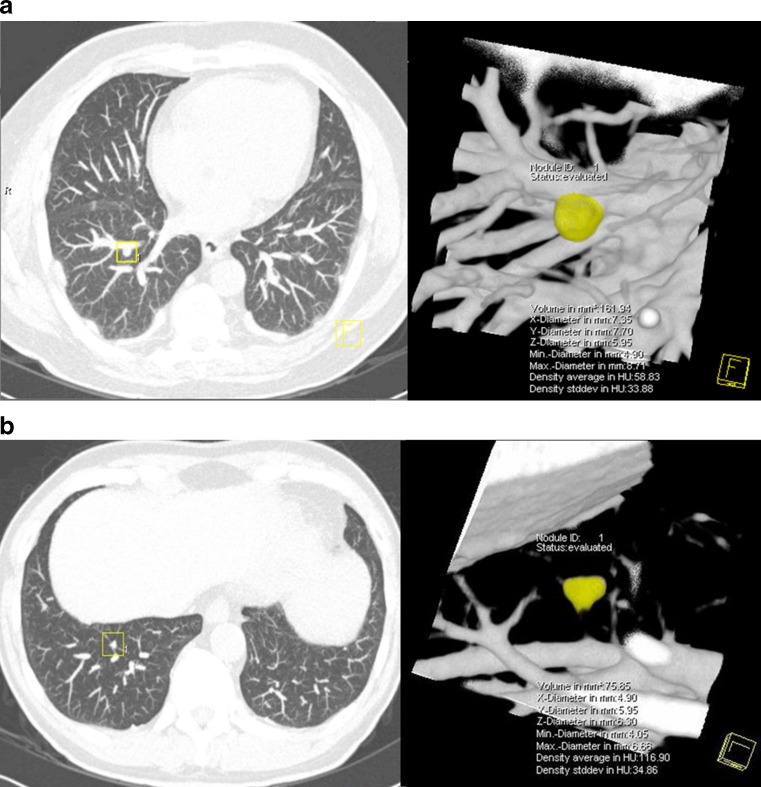
Examples of pulmonary nodules needing further evaluation that were missed by double reading. **a** Vessel-attached nodule with baseline volume 161.9 mm^3^, **b** intraparenchymal nodule with volume 75.9 mm^3^

Only 37.9 % (11/29) of vessel-attached nodules were detected by readers. A total of 69.7 % of 33 nodules not detected by readers were attached nodules, and 78.3 % of these were vessel-attached (Fig. 2). Of the non-peripheral, vessel-attached nodules, 7 out of 11 were missed by readers but all were detected by CAD. Of 33 nodules missed by readers at baseline, 24 were detected at 3-month or 1-year follow-up CT examinations. Lung cancer was diagnosed in one solid intraparenchymal nodule, found to have grown at the second-year screening CT. The baseline volume of this missed nodule was 160.7 mm^3^.

**Fig. 3:**
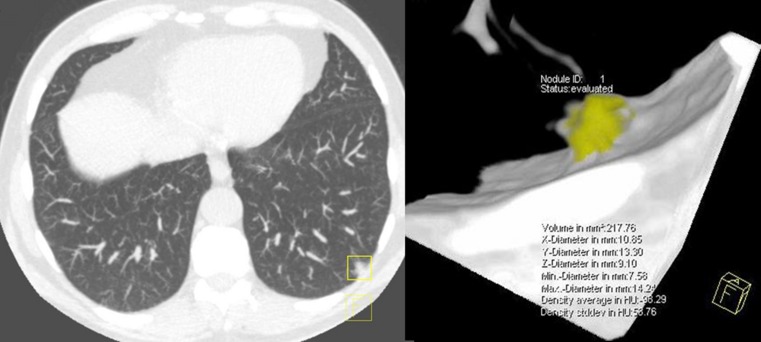
Example of a pulmonary nodule needing further evaluation that was missed by CAD: a pleural-based nodule with volume 217.8 mm^3^ (lung cancer)

One fissure-attached and two pleural-based nodules were missed by CAD. Two of five nodules missed by CAD were non-/part-solid. A solid pleural-based nodule with volume 217.8 mm^3^ missed by CAD was diagnosed as lung cancer after it was found to be growing on the 3-month follow-up CT examination, with volume doubling time less than 400 days (Fig. 3).

Through the fourth screening round (the 7th year), in total 7 lung cancers (all adenocarcinomas) have been diagnosed. None of the lung cancers originated from FP results from CAD. Three of the cancers were proven by biopsy during the baseline round, 3 during the second screening round, and 1 at the fourth round screening. None of the benign-appearing pulmonary nodules presented with malignant behaviour during subsequent screening rounds.

## Discussion

Of the 1,667 findings on lung cancer screening CT by readers and CAD, presented to the consensus panel, 90.9 % could be excluded from further evaluation with small size of pulmonary nodules being the main reason (49.2 %). The false-positive findings by CAD decreased from 3.7 to 1.9 per CT by using a nodule volume cut-off (larger than 50 mm^3^). Given the 151 (9.1 %) findings that needed further evaluation, 33 nodules (21.9 %) would have been missed if CAD was not applied. The sensitivity of nodule detection by readers could have increased by 18.6 % (from 78.1 % to 96.7 %) if CAD had also been used. However, only one lung cancer missed by readers was detected by CAD.

Numerous studies have demonstrated that the introduction of CAD in radiological practice can significantly improve the diagnostic accuracy of pulmonary nodule detection. The reported sensitivity of CAD ranged from 38 to 100 % [28–35]. Our study indicates a high sensitivity of greater than 95 % when LungCAD software is used. By using CAD, an extra 18.6 % of nodules were detected. A general comparison between our study and previous studies is, however, not possible due to the differences in methods, e.g. regarding the CT technique and the threshold of nodule size for CAD. These types of differences may explain the wide range in sensitivity reported.

High FP rate is still a considerable drawback of CAD. In this study, the FP rate of CAD was low compared to that of other studies (range 1.3–13.4/case, average 4.7/case) [28–35]. However, it is still higher than the FP rate for double reading (0.5/case). Over 80 % of the FPs in this study were reported only by CAD, of which nearly half could be excluded from further evaluation if nodule size was considered. Using a nodule volume cut-off of greater than 50 mm^3^, the FP rate decreased to 1.9 per CT. The use of 50 mm^3^ (equal to 4.6 mm diameter for a spherical nodule) as cut-off volume for pulmonary nodules is supported by the NELSON results [14]. As published in the *New England Journal of Medicine* [14], the NELSON nodule evaluation protocol with a negative screening result in case of nodules smaller than 50 mm^3^ had a sensitivity for lung cancer of 94.6 %, whereas none of the scarce interval cancers between the first and second year screening were due to malignancy in pulmonary nodules smaller than 50 mm^3^. The chance of finding lung cancer in a participant on a second-round screening CT after a negative baseline test was only 1 in 1,000, confirming the safety of the current approach and the negligible 1-year risk of lung cancer in very small pulmonary nodules (smaller than 50 mm^3^). Use of CAD led to one additional lung cancer being detected, whereas one malignant pulmonary nodule was missed by CAD. Both nodules had a volume greater than 50 mm^3^.

Among all FP findings identified by CAD, nearly 40 % were considered benign lesions by the consensus panel, e.g. fissure thickening and pleural plaque. In a previous study by Wormanns et al. [35] concerning nodules adjacent to the pleural surface, none of the 21 pleural-based findings detected by CAD were regarded as true pulmonary nodules. Given the high rate of this type of CAD finding in our study, one may conclude that CAD has difficulty in distinguishing pleura-based nodules and pleural plaques. This may be caused by the image segmentation component of the algorithm which may regard a part of the chest wall as a nodule and include it in further image processing. On the other hand, the one lung cancer missed by CAD was a pleural-based nodule. A considerable number of FP findings for CAD concerned vessels and rib bulging which were frequently misinterpreted owing to their nodule-like appearance in cross sections. Another principal problem was the difficulty in establishing a density value as threshold for lesion detection as a result of partial volume effects and motion artefacts. All non-lesions were easily distinguished from nodules by the readers, particularly when 3D visualisation was used in the pulmonary nodule evaluation platform.

Various factors affect nodule recognition during screening including reader experience and variability, CT technique and viewing conditions, as well as nodule characteristics [36]. The performance of readers can be influenced by nodule location and its relationship to surrounding anatomical structures [37, 38]. The radiologist has little difficulty in finding peripheral and subpleural nodules even if they are small because there are no vessels of similar size near the pleural surface [39]. In central lung regions, however, nodules can go undetected because they can be confused with blood vessels imaged in axial cross sections [35, 40]. A lesion not noticed by a reader because of a particular location, may often be detected in retrospective review after being detected on a subsequent CT. We found that vessel-attached nodules in particular can be missed by human readers. Although Marten et al. [41] reported that readers recognised more of the nodules with vascular attachment, Naidich et al. [37] showed that nodules either overlapping or superimposing blood vessels were harder for radiologists to identify (sensitivity 32.5 %). In our study, 30.3 % of the attached nodules were not detected by human readers, and 78.3 % of these missed nodules were vessel-attached. Furthermore, the mean size of vessel-attached nodules missed by readers was larger than that of other subtypes (data not shown). This indicates that contact with vessels increases the difficulty of detection by radiologists. The study by Naidich [37] demonstrated a significant relationship between nodule location and detectability by human readers (sensitivity: peripheral 73.9 %, central 48.6 %, perihilar 36.7 %). Of the non-peripheral nodules, two-thirds were found by double reading, considerably higher than in the aforementioned article. However, of the non-peripheral, vessel-attached nodules, 7 out of 11 were missed by readers. CAD was significantly more sensitive for these types of nodules detecting all 11 of them.

In our study, the percentage of sub-solid nodules was low (5 nodules, 3.3 %), similar to the relatively low prevalence in our entire lung cancer screening study (2 %) [26]. Two of the five non-/part-solid nodules were missed by CAD but none were missed by readers. Most CAD systems so far are designed and optimised for solid nodules. The obstacle of adequate detection of sub-solid nodules is primarily caused by the setting of an attenuation range. The selection of texture features will affect the diagnostic performance of the final CAD scheme [42]. In a small study by Armato [40], four of six lung cancers not detected by automated detection were non-solid and two were part-solid. A computerised scheme based on the application of artificial neural networks to selected texture features and Gaussian curve fitting features may hold promise for facilitating detection of localised sub-solid nodules in CT [42]. The CAD used in our study does not support the detection of nodules with non-solid components. Furthermore, the number of sub-solid nodules was small. Evaluation of the benefit of new CAD systems with improved sensitivity for sub-solid nodules should be conducted in future studies with larger numbers of sub-solid nodules.

A limitation of our study is that nodule diagnosis was in most cases (intermediate-sized nodules) not directly proven by biopsy but by evaluation of nodule growth on a short-term follow-up CT examination. However, the aim of the current study was to assess the performance of CAD versus double reading by human readers for detecting potentially relevant pulmonary nodules, which is the first step on the road to diagnosing early stages of lung cancer. The reference standard for defining the presence of a pulmonary nodule was an experienced consensus panel. Reader experience and variability could have affected the results. However, by using the interpretation of the sum of all findings by a consensus panel as the reference standard the effect of reader variability was reduced if not minimised. Also, the consensus panel did not perform a free search for potential additional findings. It is theoretically possible that the consensus panel could have found one or more additional pulmonary nodules. However, in view of the extremely high sensitivity of CAD for pulmonary nodules [32, 34], this was considered unlikely. As a result of the small numbers of certain nodule types, logistic regression could not be reliably performed for all nodule characteristics. Although we have demonstrated the benefits of CAD complementary to double reading compared to double reading alone, timing of the two modes still can be investigated; this actually depends on the efficiency of the workflow for CAD mark review. Lastly, the current analysis was based on a certain type of CAD software and a specific protocol for double reading and nodule evaluation. Whether the results can be generalised to other types of CAD software was not determined; however, the results are in line with previous reports on the improved sensitivity of pulmonary nodule detection by CAD compared to that of human readers [15, 32].

In conclusion, using a combination of CAD and nodule size cut-off in lung cancer screening improves the sensitivity of pulmonary nodule detection compared to that of double reading, without missing lung cancers. Adding a nodule volume cut-off of 50 mm^3^ to CAD leads to nearly half the FP rate (1.9 versus 3.7 FP/CT) with an increase in positive predictive value.
